# The Red Queen Gets a New Lease on Life

**DOI:** 10.1371/journal.pbio.0040285

**Published:** 2006-08-01

**Authors:** Liza Gross

Why does sex exist? A long-popular view holds that sexual reproduction creates new gene combinations that help the next generation resist rapidly co-evolving parasites. Each species constantly changes to achieve the same result—evolutionary advantage—prompting evolutionary biologists to dub this hypothesis the Red Queen (who tells Alice in *Through the Looking Glass* “it takes all the running you can do, to keep in the same place”).

Recent theoretical studies have challenged the generality of the Red Queen hypothesis, suggesting that even though parasites can exert selection pressures that favor sex under some conditions, more often they select against it. They do this, the studies found, by selecting against genes that increase the degree of genetic mixing. And now, Aneil Agrawal has come to the Red Queen's rescue with his own theoretical analysis. While the recent models assumed that host–parasite encounters are random, Agrawal shows that when nonrandom interactions are assumed—so that a host is more likely to acquire parasites from its mother—selective pressures from parasites are much more likely to favor sex.

In theoretical models that assume random host–parasite interactions, the host's fitness depends only on its own genetic makeup (or genotype), a scenario called genotypic selection. If the host stands a reasonable chance (above what would be expected to occur randomly) of contracting infection from its mother, host fitness will also depend on the host's genetic similarity to its mother (called similarity selection).

Agrawal's model allows an individual host's fitness to depend both on its own genotype and on its similarity to its mother's genotype. This framework can describe selection by parasites that are encountered randomly or are transmitted by the mother. Risk of maternal transmission will be high when parasites pass directly from mother to offspring through her eggs. The likelihood of transmission will diminish if offspring acquire infection after dispersal. Offspring that have the same genotype as their mother will be more susceptible to parasites from their mother than those with different genes. Thus, Agrawal explains, to the extent that maternal transmission occurs, hosts will be subject to both genotypic and similarity selection.

If the Red Queen hypothesis is true, and host–parasite co-evolution underlies the evolution and maintenance of sex, then these species interactions should create links between gene variants (or alleles) that enhance genetic mixing and alleles related to fitness. (The alleles that influence genetic mixing are called modifier alleles, because they influence the degree of investment into sexual rather than asexual reproduction.)

Agrawal first determined how a modifier allele evolves under different scenarios involving genotypic and similarity selection. He then evaluated the extent of genotypic and similarity selection produced by host–parasite co-evolution, and showed how the likelihood of maternal transmission affects whether parasites select for or against sex. He found that even though similarity selection has a much weaker effect than genotypic selection on fitness, it can exert a powerful force on the evolution of modifier alleles (and thus sex). Even a small chance of maternal transmission can lead to parasite selection for sex, Agrawal explains, because similarity selection affects genetic associations between mother and offspring, which tend to be strong (as opposed to genetic associations within offspring, which tend to be weaker).

Previous models have shown that sex is favored under very limited conditions in large, randomly breeding populations because genetic mixing tends to break down beneficial gene combinations produced by selection, which presumably enhance fitness. By incorporating the fitness effects of similarity selection, Agrawal could examine similarity selection's potential impacts on the evolution of modifier alleles independent of its fitness effects—and discover that parasites are “much more likely to favor sex.” The model predicts that this is most likely to occur when parasites are directly transmitted from mother to offspring, virulence is low, and infection rates are high (otherwise, too few offspring are produced by infected mothers).

While Agrawal doesn't argue that parasites fully explain why sex evolved, his results show that accounting for real-world transmission scenarios puts the ball squarely back in the Red Queen's court. Researchers can use his model to study the evolution of sex under a wide range of scenarios, such as when individual fitness depends on kin or other social groups.

## 

**Figure:**
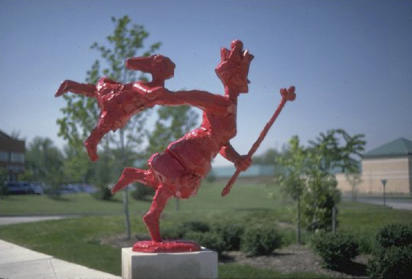
The Red Queen hypothesis posits that sex allows hosts to evade co-evolving parasites. (Photo: William F. Duffy)

